# Orthostatic responses in adolescent chronic fatigue syndrome: contributions from expectancies as well as gravity

**DOI:** 10.1186/1751-0759-8-22

**Published:** 2014-09-15

**Authors:** Vegard Bruun Wyller, Even Fagermoen, Dag Sulheim, Anette Winger, Eva Skovlund, Jerome Philip Saul

**Affiliations:** 1 Department of Pediatrics, Oslo University Hospital, N-1478 Oslo, Norway; 2 Division of Medicine and Laboratory Sciences, Medical Faculty, University of Oslo, Oslo, Norway; 3 Department of Pediatrics, Akershus University Hospital, Nordbyhagen, Norway; 4 Institute of Clinical Medicine, Medical Faculty, University of Oslo, Oslo, Norway; 5 Department of Anesthesiology and Critical Care, Oslo University Hospital, Oslo, Norway; 6 Department of Pediatrics, Lillehammer County Hospital, Lillehammer, Norway; 7 Institute of Nursing Sciences, Oslo and Akershus University College of Applied Sciences, Oslo, Norway; 8 Institute of Clinical Medicine, Medical Faculty, University of Oslo, Oslo, Norway; 9 School of Pharmacy, University of Oslo, Oslo, Norway; 10 Norwegian Institute of Public Health, Oslo, Norway; 11 Department of Pediatrics, Medical University of South Carolina, Charleston, USA

**Keywords:** Adolescence, Autonomic nervous system, Chronic fatigue syndrome, Expectancies, Orthostatic intolerance

## Abstract

**Background:**

Orthostatic intolerance is common in chronic fatigue syndrome (CFS), and several studies have documented an abnormal sympathetic predominance in the autonomic cardiovascular response to gravitational stimuli. The aim of this study was to explore whether the expectancies towards standing are contributors to autonomic responses in addition to the gravitational stimulus itself.

**Methods:**

A total of 30 CFS patients (12–18 years of age) and 39 healthy controls underwent 20° head-up tilt test and a motor imagery protocol of standing upright. Beat-to-beat cardiovascular variables were recorded.

**Results:**

At supine rest, CFS patients had significantly higher heart rate, diastolic blood pressure, and mean arterial blood pressure, and lower stroke index and heart rate variability (HRV) indices. The response to 20° head-up tilt was identical in the two groups. The response to imaginary upright position was characterized by a stronger increase of HRV indices of sympathetic predominance (power in the low-frequency range as well as the ratio low-frequency: high-frequency power) among CFS patients.

**Conclusions:**

These results suggest that in CFS patients expectancies towards orthostatic challenge might be additional determinants of autonomic cardiovascular modulation along with the gravitational stimulus *per se*.

## Background

Chronic fatigue syndrome (CFS) is characterized by unexplained, long-lasting, disabling fatigue accompanied by several other symptoms [1,2]. CFS is an important cause of disability among adolescents and may have a detrimental effect on psychosocial and academic development [3], as well as family functioning [4]. Prevalence estimates vary from 0.1 to 0.5%, and more females than males are affected [5,6].

Orthostatic intolerance is a main complaint among CFS patients [1,2,7]. Accordingly, several studies have reported distinctive alterations of autonomic cardiovascular control both at supine rest and during standardized orthostatic challenge, characterized by enhanced sympathetic and attenuated parasympathetic nervous activity [8-10]. The autonomic alterations seem to be of central origin [11] and may represent a more fundamental part of the underlying CFS pathophysiology.

We have suggested that the autonomic alterations, as well as other features of CFS, might be attributed to a persistent stress response or “sustained arousal” [12], paralleling the pathophysiology of post-traumatic stress disorder [13]. The sustained arousal model complies with other recent CFS models [14] and rests upon contemporary stress theories [15-17]. Of note, this model predicts that *expectancies* modulate autonomic nervous activity. Thus, the response towards an orthostatic challenge is not only a consequence of the gravitational stimulus *per se*, but also a consequence of the expectancies towards the stimulus as well as the compensatory abilities.

The aim of the present study was to explore the differences between autonomic responses due to gravitational stimuli and autonomic responses due to expectancies in adolescent CFS. We hypothesized that expectancies might be an important determinant of the autonomic responses in CFS.

## Methods

### Participants

CFS patients were recruited from all hospital pediatric departments in Norway (n = 20), as well as primary care pediatricians and general practitioners. A diagnosis of CFS was based upon a standardized set of investigations (pediatric specialist assessment, comprehensive hematology and biochemistry analyses, chest x-ray, abdominal ultrasound, and brain MRI) carried out by the referring unit, as well as independent clinical assessment by two of the authors (EF and DS). In agreement with recent clinical guidelines [2,18] and previous studies from our group [7,9-11,19], we applied a ‘broad’ case definition of CFS, requiring three months of unexplained, disabling chronic/relapsing fatigue of new onset. We did not require that patients meet any other accompanying symptom criteria. However, we required that the patient a) was unable to follow normal school routines due to fatigue; b) was not permanently bedridden; c) did not have any concurrent medical or psychiatric disorder that might explain the fatigue; d) did not experience any concurrent demanding life event (such as parents’ divorce) that might explain the fatigue; and e) did not use pharmaceuticals (including hormone contraceptives) regularly.

Healthy controls were recruited from local schools. They were required not to have any chronic disease and not to use pharmaceuticals regularly (including hormone contraception).

### Study design

**Figure 1:**
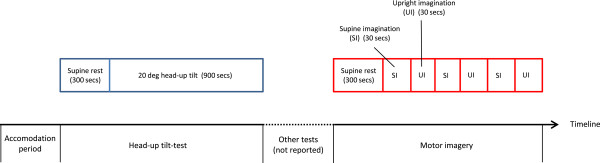
Graphical depiction of autonomic test protocols.

All participants underwent an investigational program at our research unit consisting of a one-day in-hospital assessment. Autonomic assessments that included a head-up tilt-test and a motor imagery protocol were performed around noon in a quiet room in a fixed sequence and by three researchers only (EF, DS and AW) (Figure 1). All participants were instructed to fast overnight and abstain from tobacco products and caffeine at least 48 hours in advance. Following the in-hospital assessment, a self-administered questionnaire that included questions on orthostatic intolerance was completed.

This study is part of the NorCAPITAL-project (The Norwegian Study of Chronic Fatigue Syndrome in Adolescents: Pathophysiology and Intervention Trial) (ClinicalTrials ID: NCT01040429), which encompasses a cross-sectional design, a double-blind, randomized, placebo-controlled design and a qualitative design. Details of NorCAPITAL are described elsewhere [19]. Data were collected in the period March 2010 until October 2012. Informed consent was obtained from all participants and from parents/next-of-kin if required. The study was approved by the Norwegian National Committee for Ethics in Medical Research.

This study is based upon a subset of CFS patients generated from a computer-based randomization procedure, where one fourth of the patients were randomized to be included in the present study; 18 months disease duration served as stratification criterion (cf. below).

### Autonomic assessment

Participants lay in a horizontal position and were connected to the Task Force Monitor (Model 3040i, CNSystems Medizintechnik, Graz, Austria), a combined hardware and software device for noninvasive recording of cardiovascular variables. They were allowed five minutes to accommodate to the situation, after which a 300 sec baseline registration was obtained. Thereafter, a head-up tilt-test (HUT) was performed in which participants were tilted to 20 degrees for 15 minutes using an electronically operated tilt table with a footboard support (Model 900–00, CNSystems Medizintechnik, Graz, Austria). Details of the HUT protocol have been described elsewhere [10]. The feasibility of this protocol for studying adolescent CFS patients has been demonstrated in several previous studies [9,10,19]. In particular, the low tilt angle (20°) does not normally precipitate syncope, which is otherwise a common problem among adolescents being subjected to stronger orthostatic challenges. Still, 20° head-up tilt is sufficient to demonstrate hemodynamic alterations and compensatory autonomic responses.

In a separate experiment, participants were subjected to a motor imagery protocol [20]. First, following supine rest of approximately five minutes, they were instructed to mentally simulate/imagine a situation in which they were laying supine and relaxed, for instance on a sunny beach. After 30 sec, they were instructed to imagine a situation in which they were upright, such as standing in line. Following another 30 sec, imagination of laying supine was encouraged once more. Altogether, the entire sequence was repeated twice. The motor imagery experiment was succeeded by tests of autonomic responses to other stimuli (pain, isometric exercise, nerve stretch, pictures having emotional content, and mental exercise). These tests are not reported here.

Instantaneous RR intervals and heart rate (HR) were obtained from the electrocardiogram (ECG). Continuous arterial blood pressure was obtained noninvasively using photoplethysmography on the right middle finger, a method that correlates satisfactorily with invasive pressure measurements and that is validated for adolescents and children. Mean arterial blood pressure (MBP) was calculated by numerical integration of the recorded instantaneous BP. The recorded value was calibrated against conventional oscillometric measurements of arterial BP on the left arm. Impedance cardiography was used to obtain a continuous recording of the temporal derivative of the transthoracic impedance (dZ/dt). Beat-to-beat stroke volume was calculated from the impedance signal [21]. Power spectral analysis for HRV was automatically provided by the TFM, using an adaptive autoregressive model [22]. The following indices are reported: Total Power Spectral Density (PSD), Low Frequency (LF) power (0.05 to 0.17 Hz), and High Frequency (HF) power (0.17 to 0.4 Hz), using both absolute and normalized unites. In addition, the LF/HF ratio was calculated, which is often considered an index of sympatho-vagal balance.

Data from the baseline registration, the HUT procedure and the motor imagery procedure were exported to Microsoft Excel for further calculations. Beat-to-beat stroke index (SI) was calculated by dividing stroke volume by body surface area estimated from height and weight, and beat-to-beat total peripheral resistance index (TPRI) was calculated as MBP divided by the product of SI and HR. Thereafter, the median value of all cardiovascular variables was computed in the following epochs: Baseline: 270 to 30 seconds before HUT. Early Tilt: 30 to 270 seconds after HUT. Supine Imagination: 10 to 25 seconds after the instruction was given. Upright Imagination: 10 to 25 seconds after the instruction was given. As the imagery procedure was performed three times in each individual, the mean value across three identical epochs was calculated. Finally, Delta Tilt (Early Tilt – Baseline) and Delta Imagination (Upright Imagination – Supine Imagination) were computed.

### Questionnaire

The Autonomic Symptom Profile (ASP) [23], which is a validated inventory for the assessment of autonomic symptoms, was slightly modified in order to fit our age group and distributed together with several other inventories, as described elsewhere [19]. For this study, a composite score reflecting symptoms of orthostatic intolerance was constructed from 7 single items from the ASP that address experiences of dizziness in specific situations. In the composite score, dizziness when rising from the supine/sitting position was considered the most important symptom of orthostatic intolerance and therefore scored 2 points, whereas dizziness in other situations (such as eating a strong meal, taking a hot bath/shower, etc.) was scored 1 point each. The total sum score range from 0 to 8, where higher values reflect more pronounced orthostatic problems.

### Statistical analyses

The sample size was based on a previous study in which the mean difference between CFS patients and healthy controls was in the range 0.8 – 0.9 SD for variables reflecting autonomic cardiovascular control during orthostatic challenge (such as changes in HR and diastolic blood pressure during tilt) [10]. In the present study, with samples of 30 and 39 subjects, respectively, the power is at least 80% to detect differences between groups of 0.7 SD or larger. SPSS statistical software (SPSS Inc., Chicago, Ill.) was applied for all statistical analyses. Continuous variables are reported with mean (standard deviation) or median (interquartile range), depending on the distribution; categorical variables are reported with frequencies. Statistical tests of differences between CFS patients and healthy controls were performed using Student t-test, Mann-Whitney’s test, Chi-square test or Fisher’s exact test, as appropriate. A p-value ≤ 0.05 was considered statistically significant. All tests were carried out two-sided. No correction for multiple testing was performed.

## Results

A total of 30 CFS patients and 39 healthy controls were included in this study. The two groups were comparable regarding sex, ethnicity, age, body mass index, and usage of alcohol/tobacco/narcotics (Table 1). CFS patients had a high degree of school absenteeism, but no one was permanently bed-ridden; median disease duration was 19 months.

**Table 1:** Background characteristics

	***CFS patients (n = 30)***	***Healthy controls (n = 39)***	***p-value***
Sex - no. (%)			
Male	9 (30)	11 (28)	0.871
Female	21 (70)	28 (72)	
Ethnicity – no. (%)			
Scandinavian	30 (100)	37 (95)	0.501
Not scandinavian	0 (0)	2 (5)	
Age - years, mean (SD)	15.2 (1.7)	15.2 (1.6)	0.889
BMI - kg/m^2^, mean (SD)	21.8 (4.5)	20.3 (2.9)	0.116
Alcoholic beverages - no. (%)			
Never	24 (83)	28 (78)	0.618
Occationally	5 (17)	8 (22)	
Tobacco products - no. (%)			
Never	24 (86)	29 (78)	0.450
Occationally	4 (14)	8 (22)	
Narcotics/illigal drugs - no. (%)			
Never	27 (100)	34 (92)	0.257
Occationally	0 (0)	3 (8)	
School absenteism - %, mean (SD)	64 (30)	2 (7)	<0.001
Adheres to Fukuda-criteria - no. (%)			
No	9 (31)	n.a.	n.a.
Yes	20 (69)		
Disease duration - months, median (range)	19 (56)	n.a.	n.a.

P-values are based on Chi-square test, Fisher's exact test, Student t-test or Mann-Whitney's test, as appropriate. SD = standard deviation, IQR = interquartile range, n.a. = not applicable, BMI = body mass index, Fukuda-criteria = The CFS diagnostic criteria from the International Chronic Fatigue Syndrome Study Group [1].

Symptoms of orthostatic intolerance were significantly more common in CFS patients, as reflected in the composite sum score as well as the answers to single items (Table 2).

**Table 2:** Symptoms of orthostatic intolerance

	***CFS patients***	***Healthy controls***	***p-value***
Dizzy when rising from supine or sitting position - no. (%)			
No	6 (21)	29 (81)	< 0.001
Yes	23 (79)	7 (19)	
Dizzy after eating a big meal - no. (%)			
No	26 (93)	35 (97)	0.577
Yes	2 (7)	1 (3)	
Dizzy after standing upright for a long time - no. (%)			
No	10 (35)	29 (81)	< 0.001
Yes	19 (66)	7 (19)	
Dizzy during light exercise - no. (%)			
No	10 (35)	35 (97)	< 0.001
Yes	19 (66)	1 (3)	
Dizzy during a hot bath or shower - no. (%)			
No	13 (45)	31 (86)	< 0.001
Yes	16 (55)	5 (14)	
Dizzy when seeing blood - no. (%)			
No	27 (93)	32 (89)	0.684
Yes	2 (7)	4 (11)	
Dizzy while urinating - no. (%)			
No	28 (97)	35 (100)	0.453
Yes	1 (3)	0 (0)	
Orthostatic intolerance, total score – mean (range)	3.6 (7)	0.9 (4)	< 0.001

P-values are based on Chi-square test, Fisher's exact test, or Mann-Whitney's test, as appropriate.

At supine rest, CFS patients had a higher heart rate (HR), diastolic blood pressure (DBP), and mean arterial blood pressure (MBP) and lower stroke index (SI) and heart rate variability (HRV) indices measured in absolute units (LF_abs_, HF_abs_, Total power) (Table 3). The response to 20° head-up tilt (Delta Tilt) was identical in the two groups. The response to imaginary upright position (Delta Imagination) was characterized by a stronger increase of HRV indices of sympathetic predominance (LF_norm_, LF_abs_, LF/HF) among CFS patients.

**Table 3:** Cardiovascular variables: baseline, response to 20° HUT and response to imagery upright position

	***Baseline***			***Delta tilt (response to 20° HUT)***			***Delta imagination (response to imagery upright position)***		
	***CFS patients***	***Healthy controls***	***p-value***	***CFS patients***	***Healthy controls***	***p-value***	***CFS patients***	***Healthy controls***	***p-value***
Heart rate – beats/min, mean (SD)	81 (13)	71 (9.9)	**0.001**	4.5 (4.2)	3.2 (3.4)	0.192	1.8 (2.2)	1.4 (2.3)	0.470
Systolic blood pressure – mm Hg, mean (SD)	111 (10)	109 (9.8)	0.260	-0.18 (2.9)	0.17 (4.6)	0.701	0.37 (2.6)	0.24 (1.9)	0.819
Diastolic blood pressure – mm Hg, mean (SD)	69 (7.6)	64 (7.2)	**0.017**	1.5 (2.4)	1.8 (4.2)	0.783	0.25 (1.6)	0.31 (1.8)	0.886
Mean arterial blood pressure – mm Hg, mean (SD)	84 (8.1)	79 (7.4)	**0.025**	0.85 (2.6)	1.4 (3.9)	0.466	0.26 (1.8)	0.42 (2.0)	0.727
Stroke index – ml/m^2^, mean (SD)	47 (8.8)	51 (5.9)	**0.049**	-4.8 (4.1)	-4.5 (3.7)	0.690	-0.59 (1.6)	-0.07 (1.3)	0.162
Cardiac index – l/min/m^2^, mean (SD)	3.8 (0.59)	3.6 (0.48)	0.208	-0.17 (0.24)	-0.14 (0.23)	0.675	0.03 (0.13)	0.06 (0.10)	0.340
Total peripheral resistance index – mm Hg/l/min/m^2^, mean (SD)	7.9 (1.6)	8.6 (1.9)	0.099	0.55 (0.78)	0.67 (1.0)	0.587	-0.06 (0.37)	-0.14 (0.39)	0.397
LF_norm_ – nu, mean (SD)	51 (19)	51 (15)	0.953	8.3 (9.8)	6.7 (12)	0.560	0.64 (4.7)	-1.4 (3.3)	**0.042**
HF_norm_ – nu, mean (SD)	49 (19)	49 (15)	0.953	-8.2 (9.7)	-6.8 (12)	0.579	-0.64 (4.7)	1.4 (3.3)	**0.042**
LF_abs_ – ms^2^, median (IQR)	457 (411)	884 (770)	**0.001**	-36 (409)	-92 (675)	0.753	1.9 (66)	-34 (99)	**0.026**
HF_abs_ – ms^2^, median (IQR)	440 (659)	766 (967)	**0.012**	-266 (888)	-370 (1019)	0.952	-0.9 (64)	-18 (91)	0.628
LF/HF – median (IQR)	0.97 (1.8)	1.1 (0.93)	0.942	0.16 (0.60)	0.20 (0.55)	0.818	0.004 (0.19)	-0.07 (0.14)	**0.025**
Total power - ms^2^, median (IQR)	1092 (1415)	2172 (2260)	**0.001**	-296 (1014)	-320 (2278)	0.952	-28 (131)	-64 (207)	0.107

P-values are based on Student t-test or Mann-Whitney's test, as appropriate. SD = standard deviation, IQR = interquartile range. LFnorm = Heart rate variability in the low-frequency range, normalized units; HFnorm = Heart rate variability in the high-frequency range, normalized units; LFabs = Heart rate variability in the low-frequency range, absolute units; HFabs = Heart rate variability in the high-frequency range, absolute units. Bold numbers indicate p ≤ 0.05.

## Discussion

This study shows that CFS adolescents are burdened by symptoms of orthostatic intolerance. Their baseline cardiovascular variables as well as response to imagery of upright position is significantly different from healthy controls; however, their response to head-up tilt is not. We speculate that their expectancies towards orthostatic challenge might be important determinants of autonomic cardiovascular control in addition to the gravitational stimulus *per se*.

The group differences at baseline confirm findings from several previous reports [7,10,24,25]. Increased MBP directly suggests an altered set-point of the baroreceptor-reflex. HR increases correspondingly due to enhanced sympathetic and/or attenuated parasympathetic cardiac control, as reflected in the differences of the HRV indices. The lower SI in the CFS group might be a consequence of increased HR and reduced filling time [26]. Taken together, these results are congruent with different expectancies among CFS patients and controls towards the experimental procedures following immediately after the baseline registration period. If these procedures are perceived as more stressful for CFS patients, as suggested from the sustained arousal-model, an alteration of reflex set-point and a centrally driven enhancement of sympathetic nervous activity is to be expected [15]. Alternatively, the group differences at baseline might be a consequence of sedentary deconditioning [27,28]. It should be noted, though, that none of the CFS patients was permanently bedridden.

Motor imagery activates the autonomic nervous system due to central processes; the magnitude of activation is proportional to the simulated effort [20,29]. To the best of our knowledge, autonomic activation during motor imagery has never been explored in CFS patients. In this study, during imagination of standing, CFS patients had a slight increase in RRI-variability in the LF-band, a corresponding decrease of RRI-variability in the HF-band, and a slight increase in the LF/HF-ratio. In contrast, healthy controls decreased in LF-power and LF/HF-ratio. In both groups, the changes from baseline are rather subtle. Still, the between-group differences in HRV indices indicate a predominance of sympathetic cardiac control in the CFS group [30], suggesting that CFS patients anticipated standing to be more challenging than did healthy controls. The underlying reason for this anticipation might be their previous experiences of orthostatic symptoms.

During head-up tilt, blood tends to pool in the legs due to gravity; the associated unloading of the cardiopulmonary receptors activates compensatory reflexes characterized by increased HR and total peripheral resistance index (TPRI) [15]. In this study, the cardiovascular responses were similar among CFS patients and healthy controls, whereas previous studies using an identical tilt protocol found significant differences between the two groups [10,24]. The reasons for this discrepancy are not clear; however, it is the control group responses rather than the CFS group responses that seem to deviate most from previous findings.

Other limitations of this study include a relatively small number of participants. The wide diagnostic criteria of CFS patients might have obscured results relevant to subgroups: however, similar criteria have been successfully applied in previous studies of autonomic responses in CFS patients [10,24]. Furthermore, the study design did not allow a detailed exploration of the relationship between imagery responses, gravitational responses and symptoms; thus, we are unable to tell whether expectancies may cause the CFS patients’ frequent experiences of orthostatic intolerance.

## Conclusion

CFS adolescents have symptoms of orthostatic intolerance and a different autonomic cardiovascular control at baseline as well as during imagery upright position as compared to healthy controls; however, their response to HUT is almost identical. Thus, the CFS patients’ expectancies towards orthostatic challenge might be an important and previously under-emphasized determinant of autonomic cardiovascular modulation along with the obvious determinant of the gravitational stimulus *per se*. The impact of expectancies on other CFS phenomena might be an important area for further research.

## Abbreviations

ASP: Autonomic symptom profile; CFS: Chronic fatigue syndrome; CDC: Centers for disease control and prevention; DBP: Diastolic blood pressure; HF: High-frequency; HR: Heart rate; HUT: Head-up tilt test; LF: Low-frequency; MBP: Mean arterial blood pressure; RRI: RR-interval; SBP: Systolic blood pressure; SI: Stroke index; TPRI: Total peripheral resistance index.

## Competing interests

The authors declare that they have no competing interests.

## Authors’ contributions

EF, DS and AW collected clinical data, contributed to study design and participated in data analyses. JPS contributed to study design. ES supervised data analyses. VBW conceived of the study, contributed to study design and participated in data analyses. All authors contributed to data interpretation and drafting of the manuscript. All authors read and approved the final manuscript.
